# Supplementary material for: Unraveling the phase diagram-ion transport relationship in aqueous electrolyte solutions and correlating conductivity with concentration and temperature by semi-empirical modeling
Source: Commun Chem. 2023 Sep 12;6:195. doi: 10.1038/s42004-023-00993-4 (PMC10497523; doi:10.1038/s42004-023-00993-4)

Supplementary Information (S.I)

Figure S.1: Isothermal structural variation at high and low temperatures in a phase diagram with no solvate formation

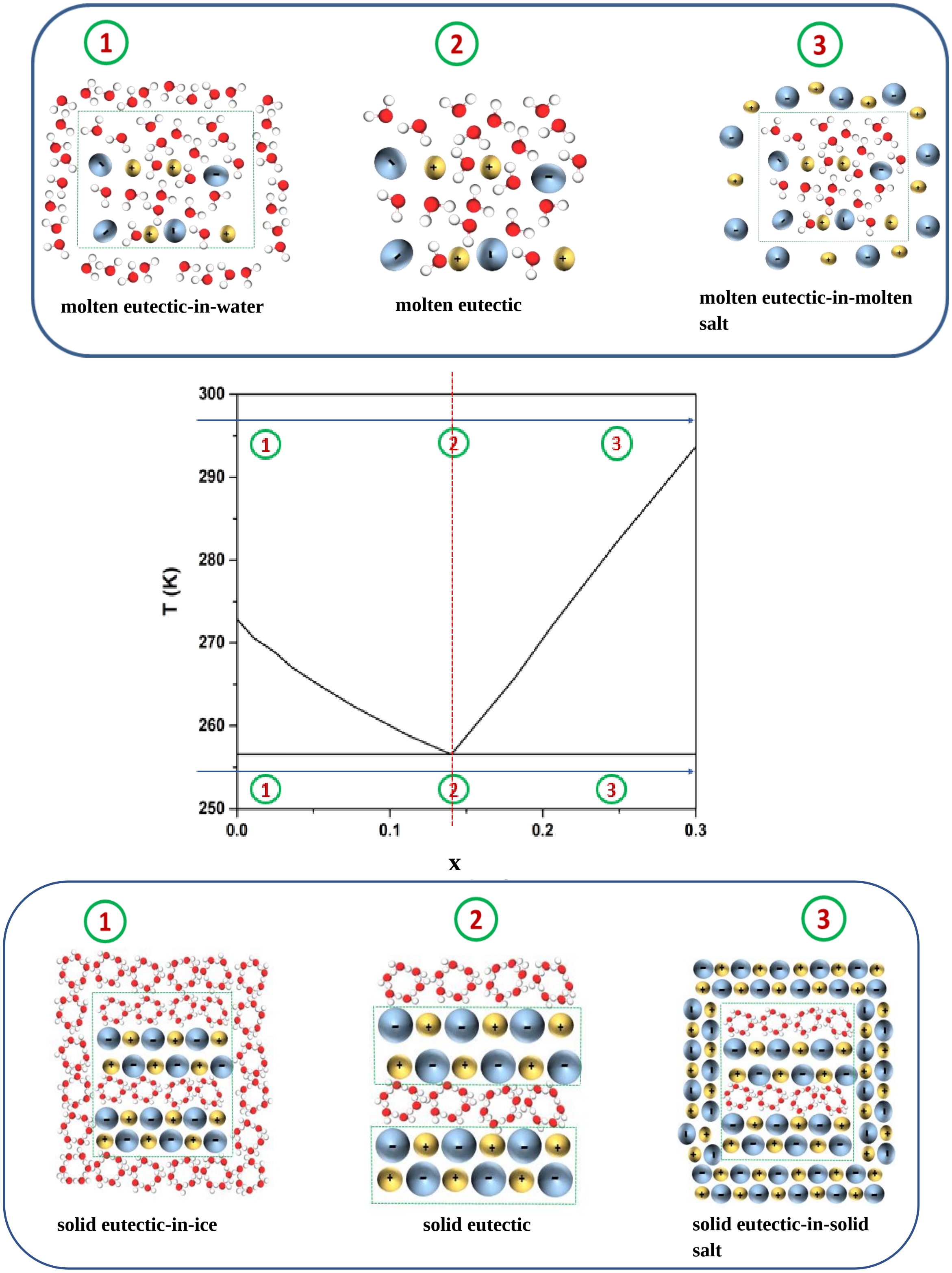

Figure S.2: Isothermal structural variation at high and low temperatures in a phase diagram with solvate formation

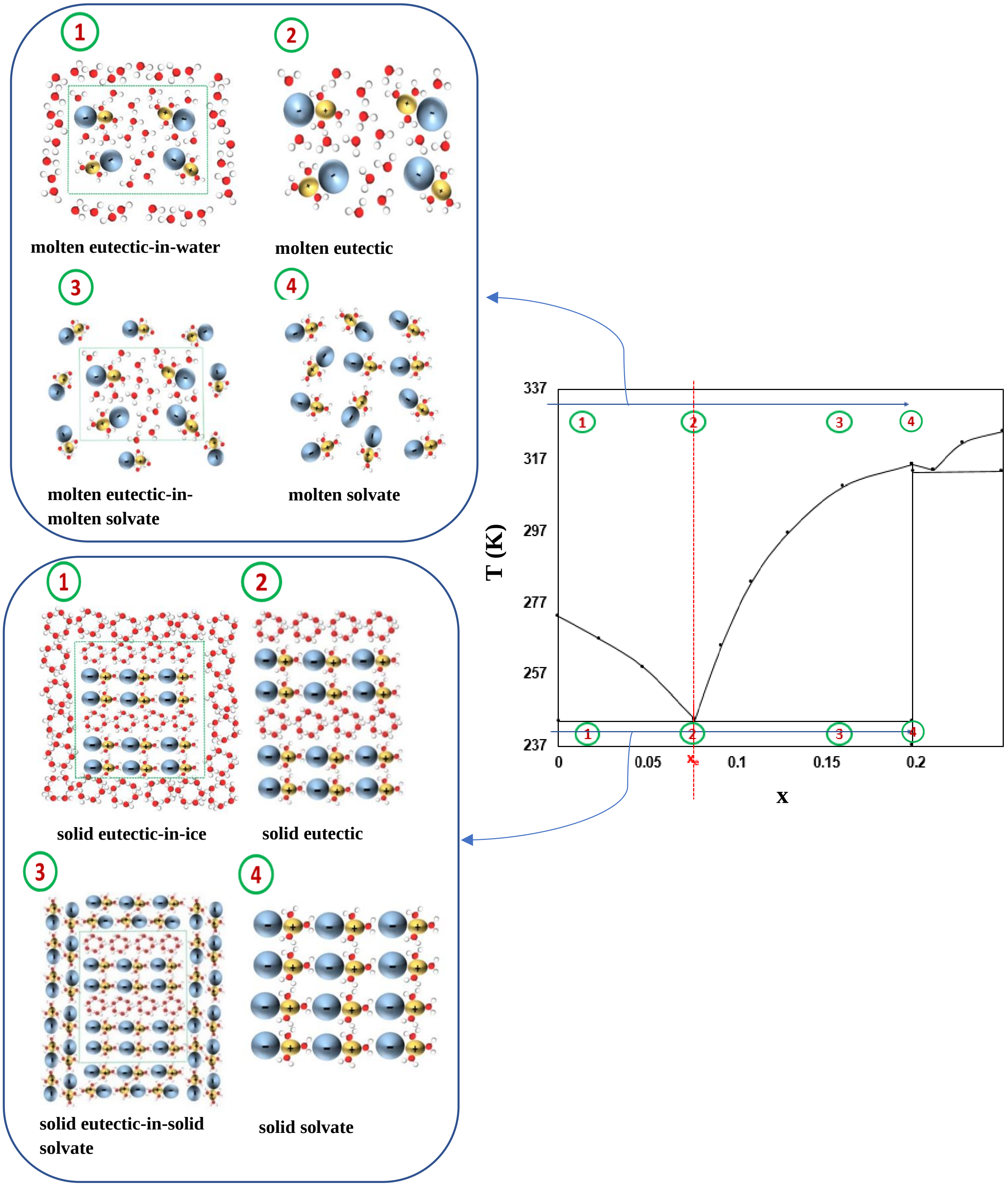

Fig. S.3: Microstructure schematic Illustration for every molar concentration/temperature

Iso-compositional structural variation in the case of:

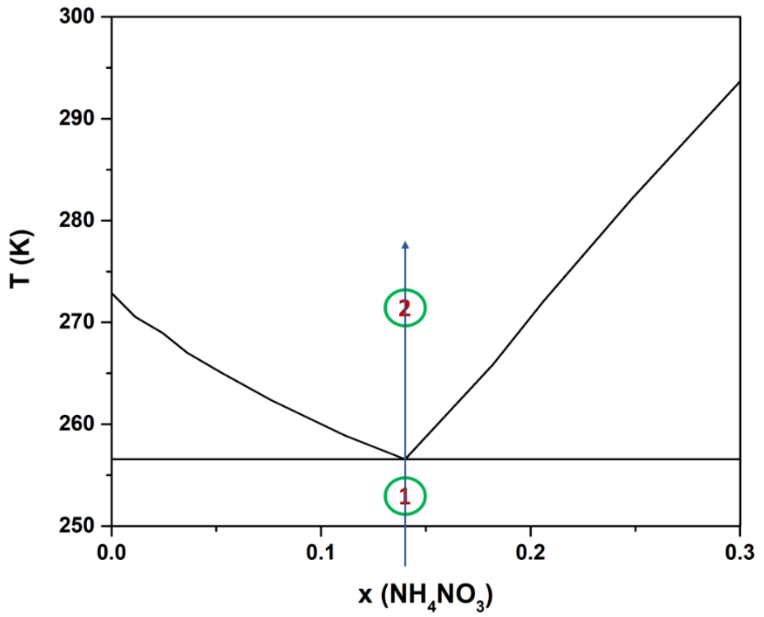

I. **Phase diagram with no solvate formation**

a. **x = x_e**

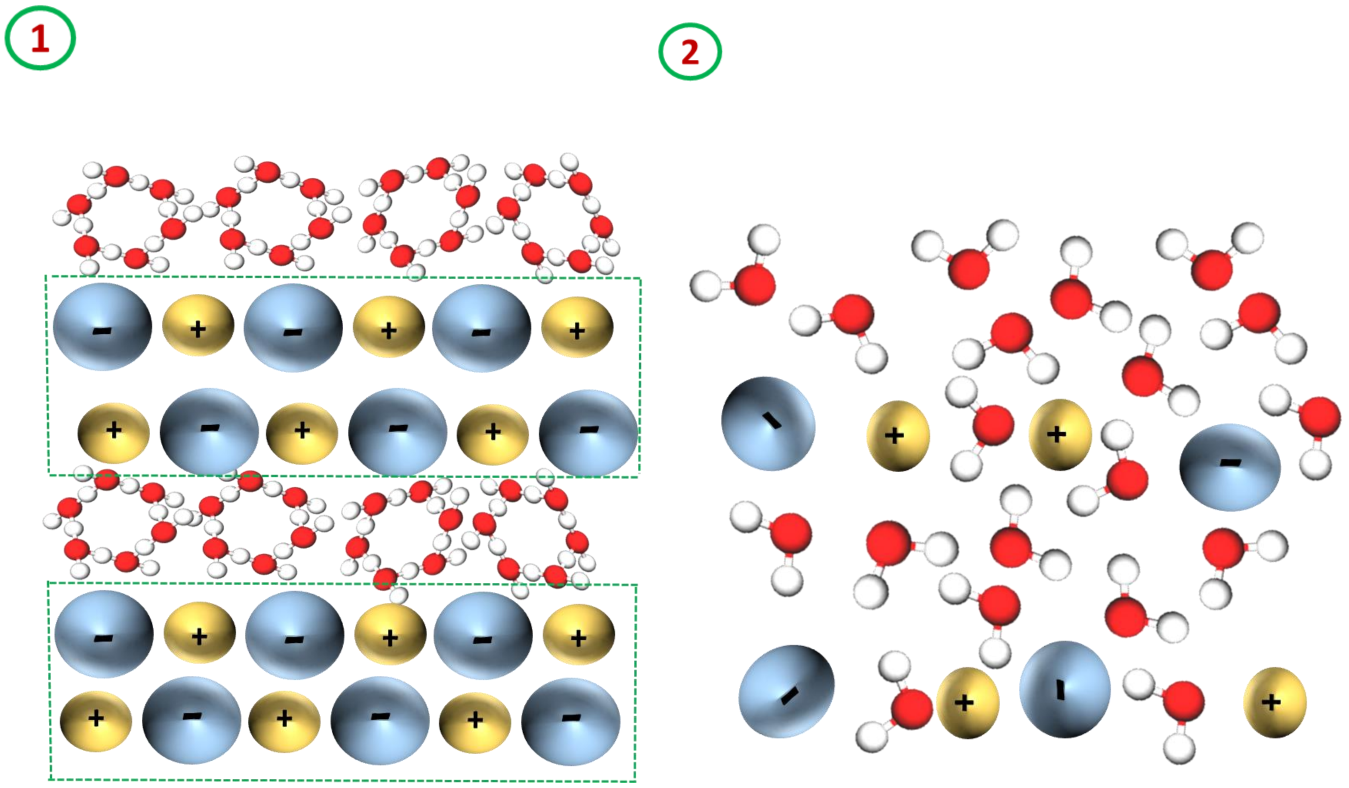

b. At $x<x_e$:

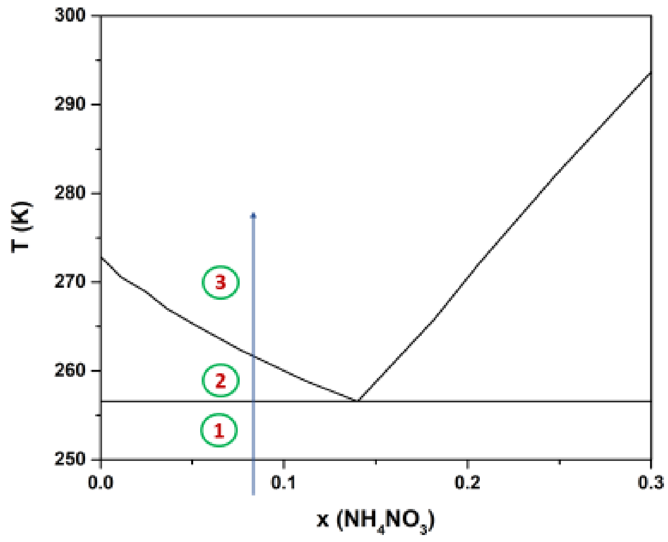

2

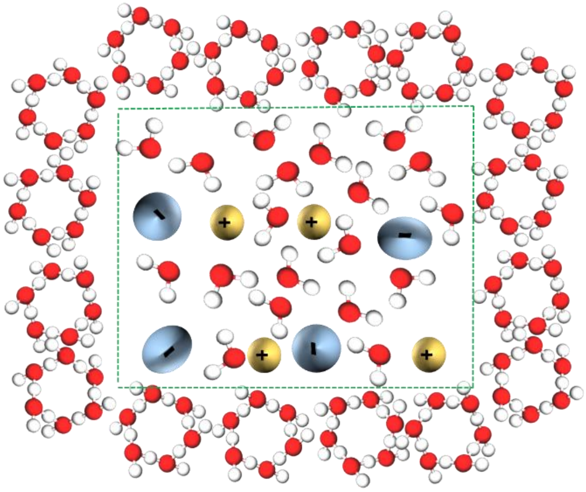

1

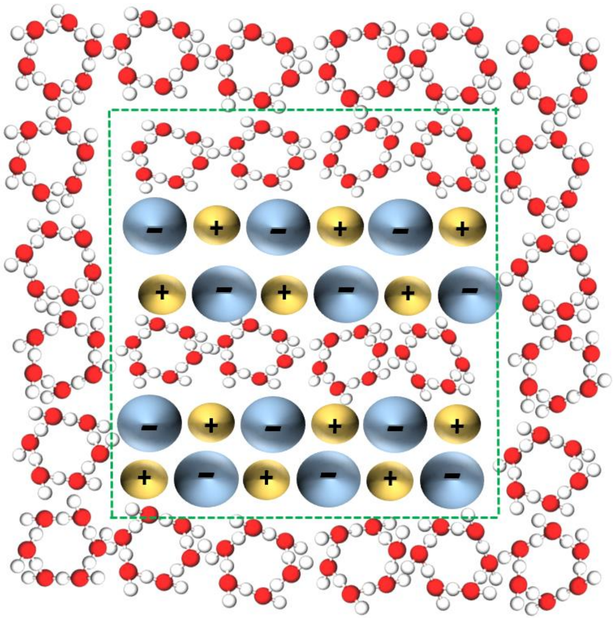

3

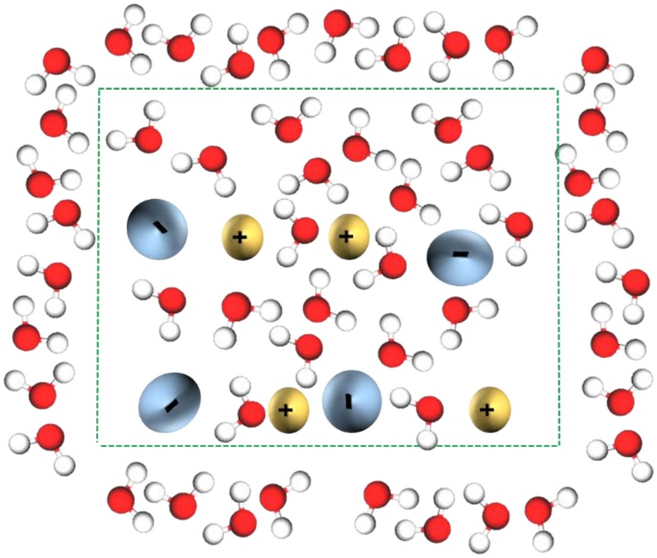

c. At $x > x_e$

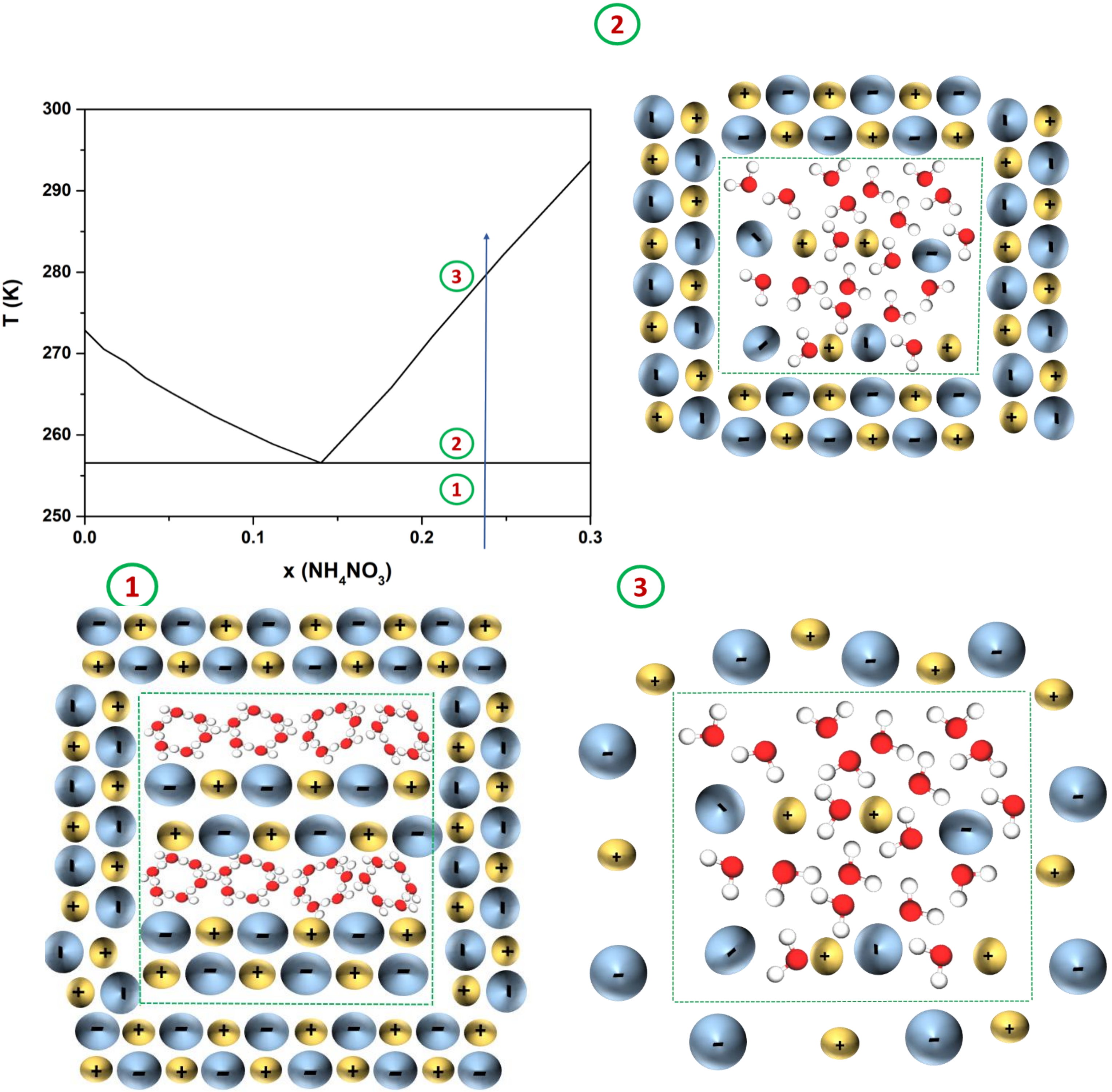

II. Phase diagram with solvate formation

a. $x = x_{e1}$

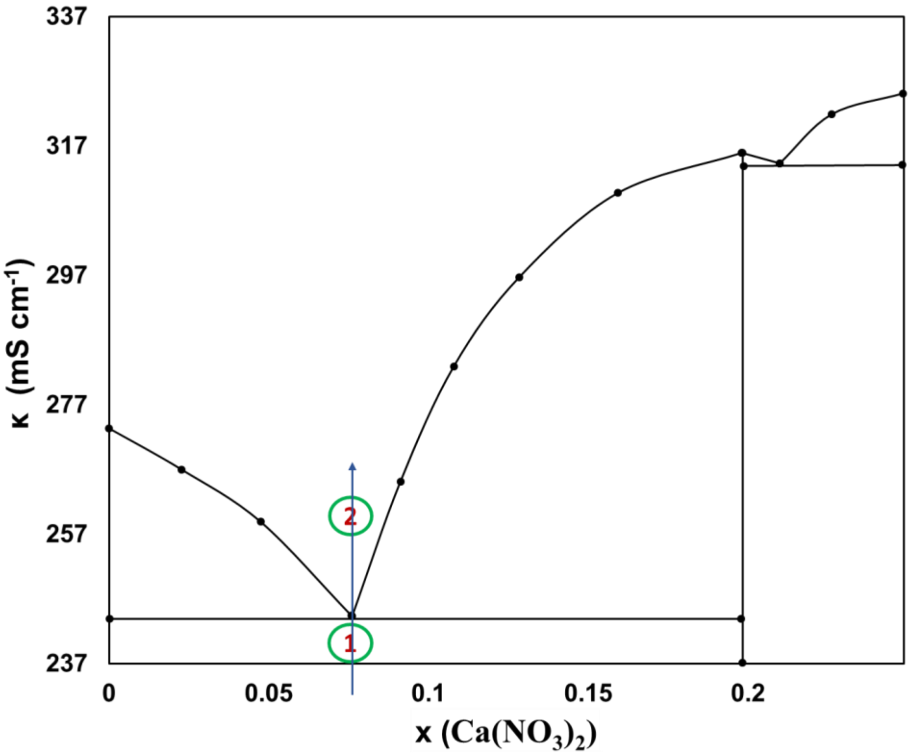

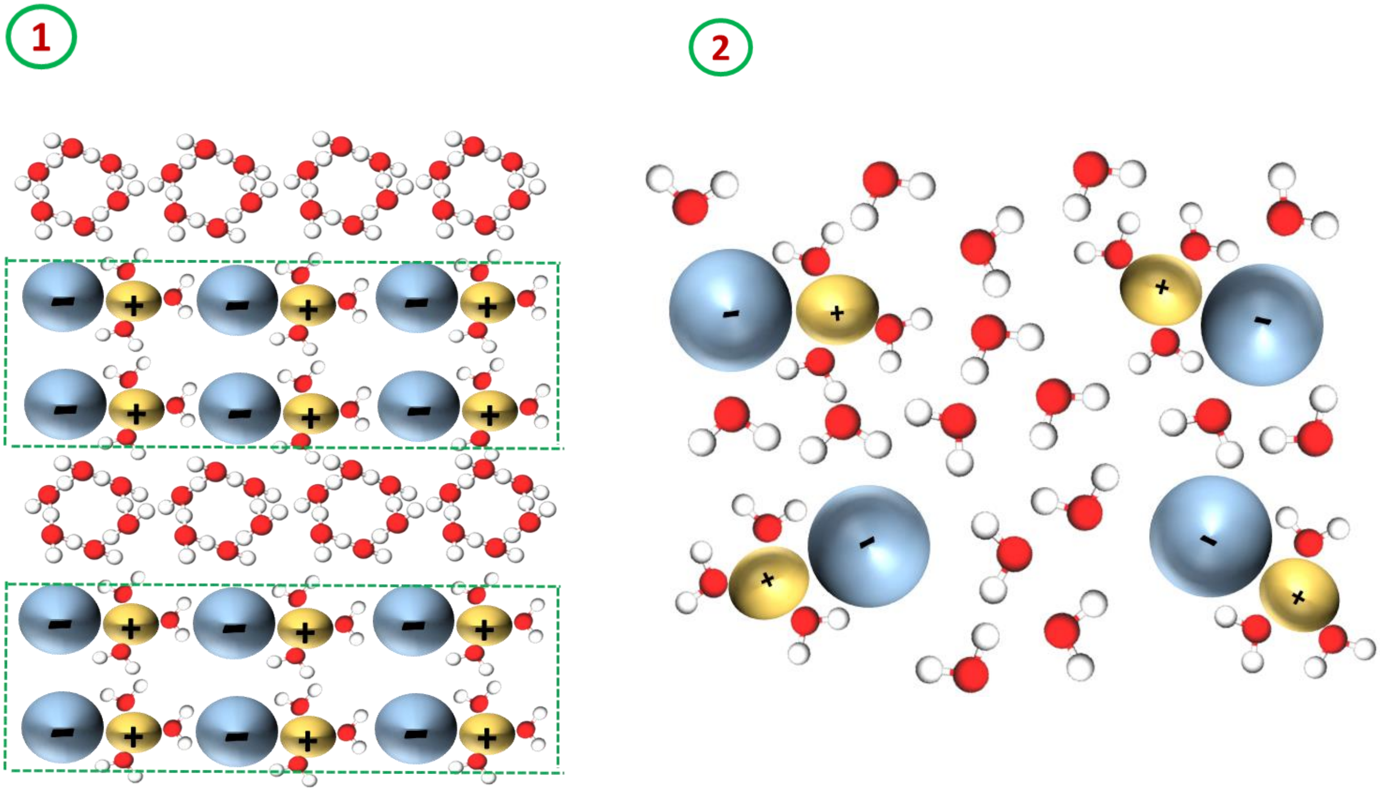

b. $x = x_S$

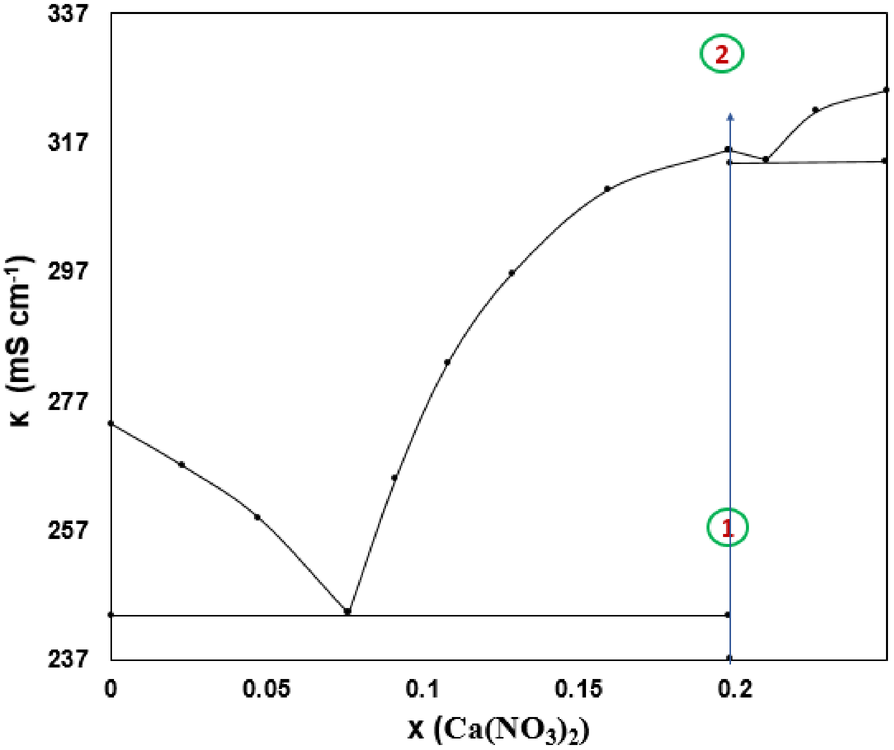

1

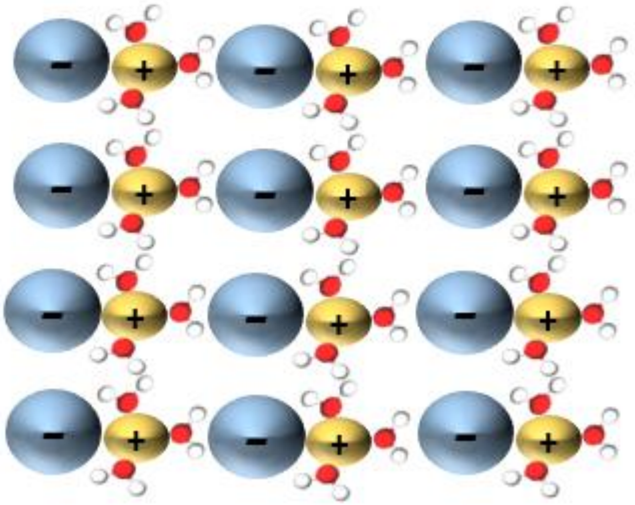

2

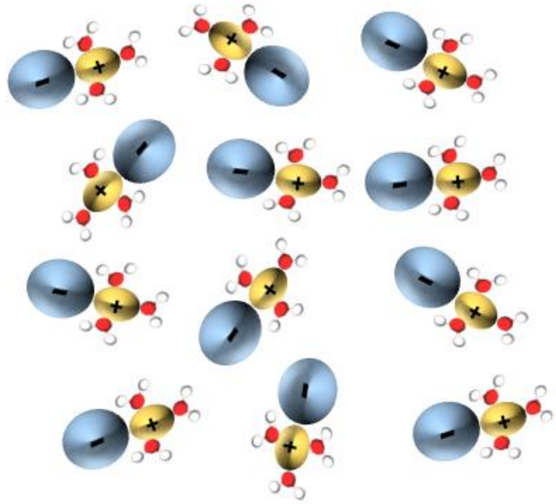

c. $x = x_{e2}$

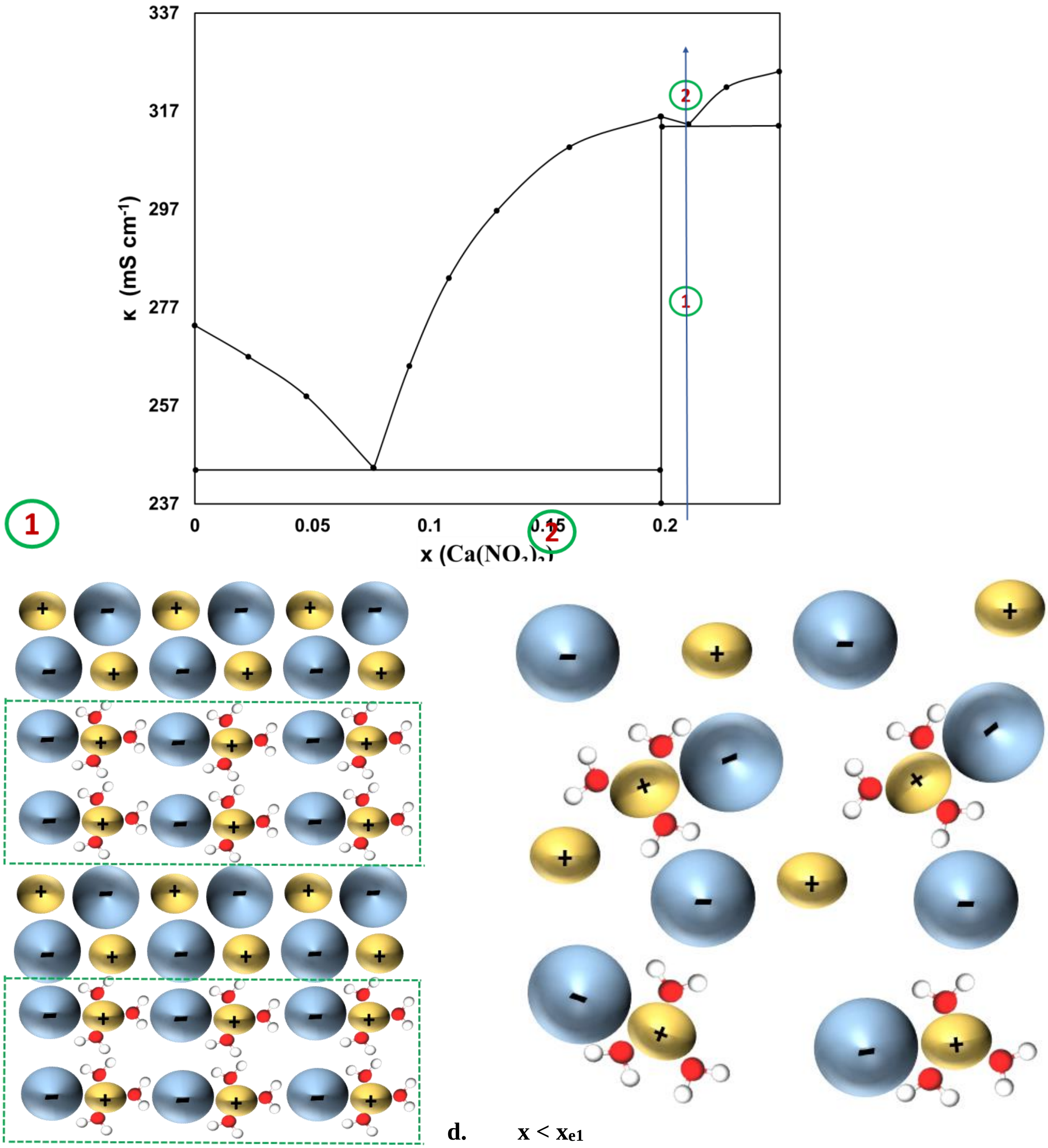

d. $x < x_{e1}$

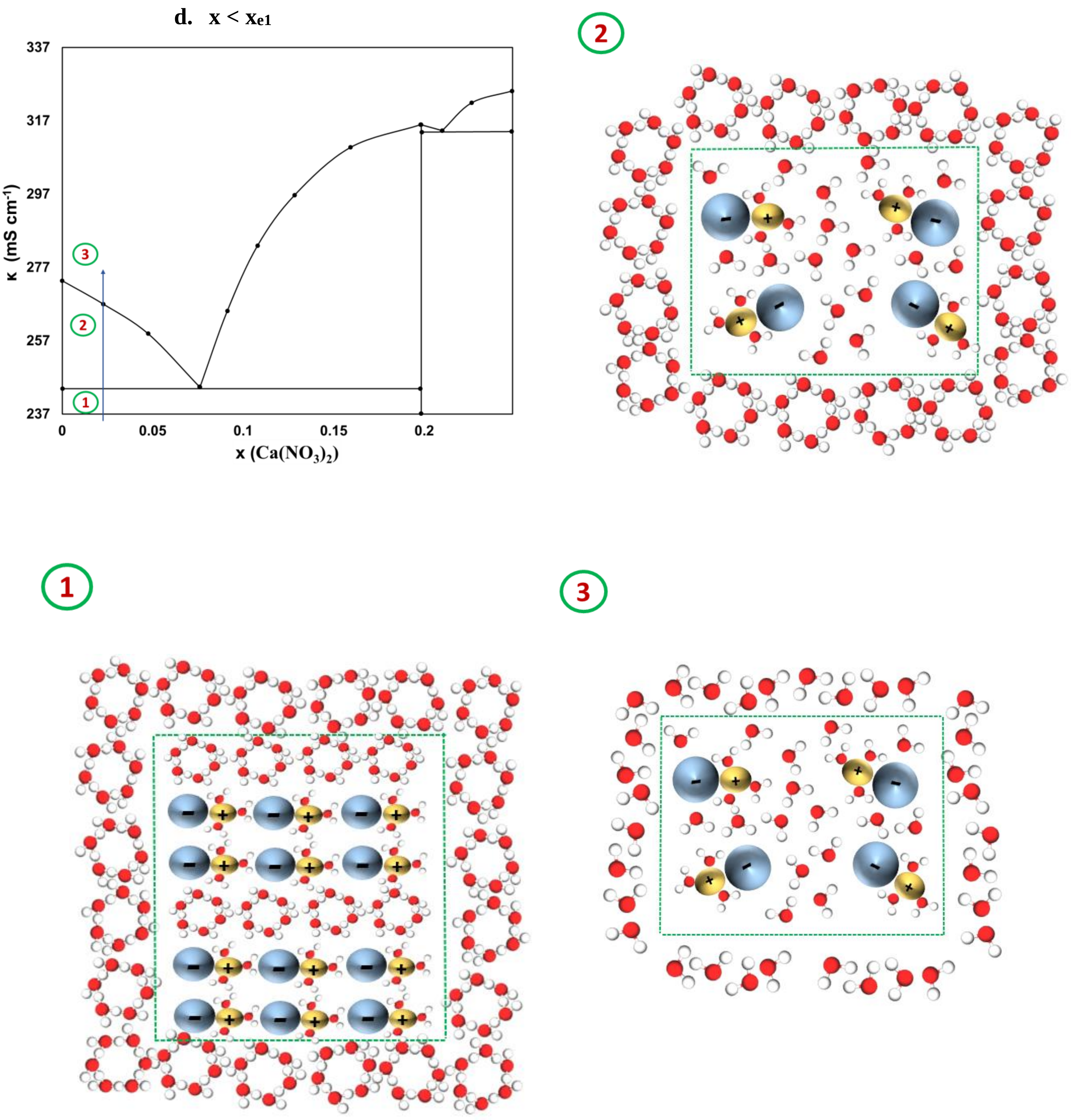

d. x < xe1
337
317
297
277
257
237
κ (mS cm-1)
0
0.05
0.1
0.15
0.2
x (Ca(NO3)2)
1
2
3

e. $x_{e1} < x < x_s$

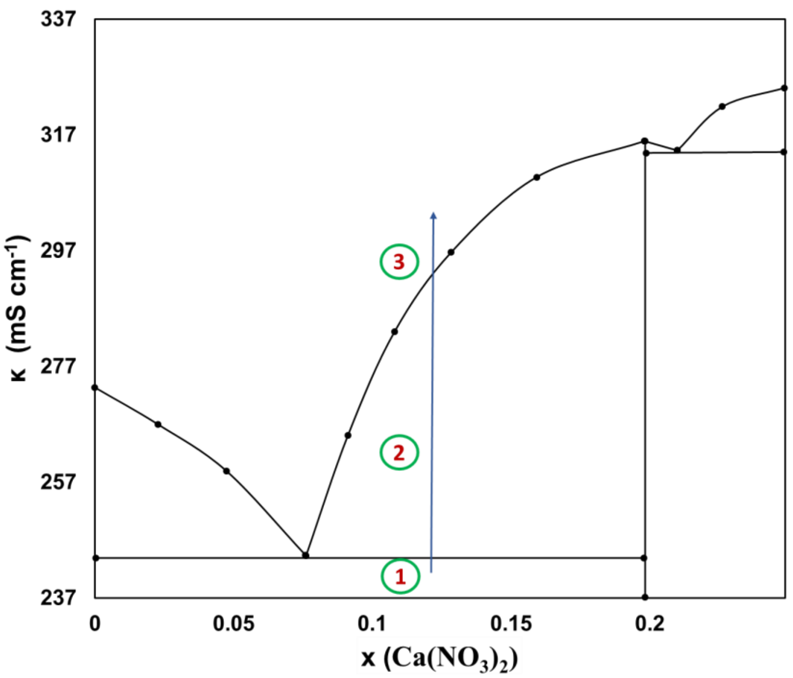

2

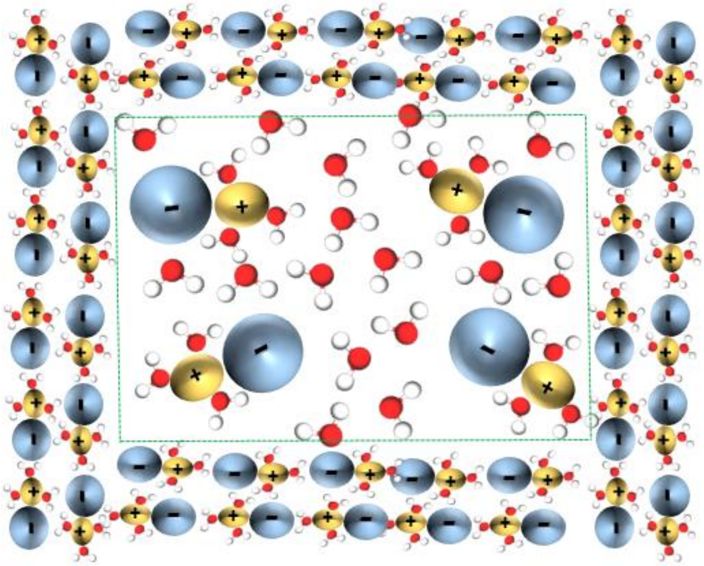

1

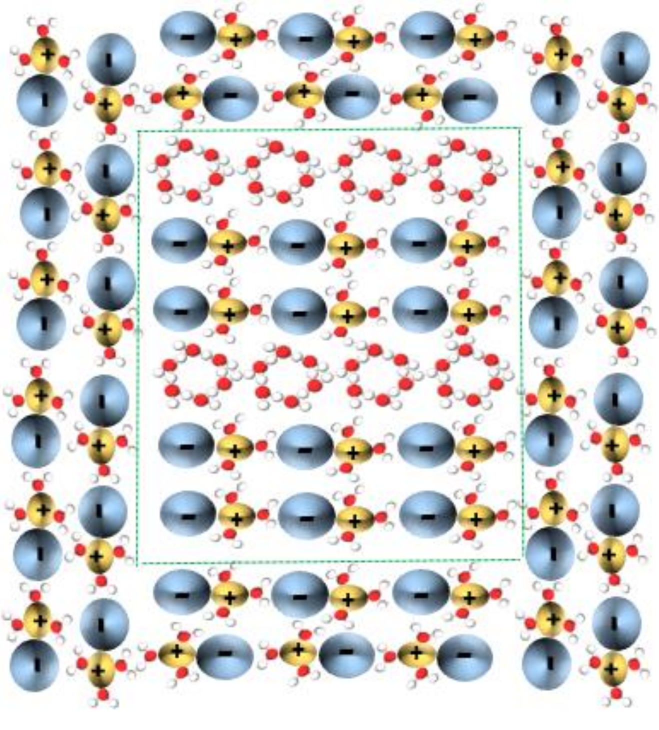

3

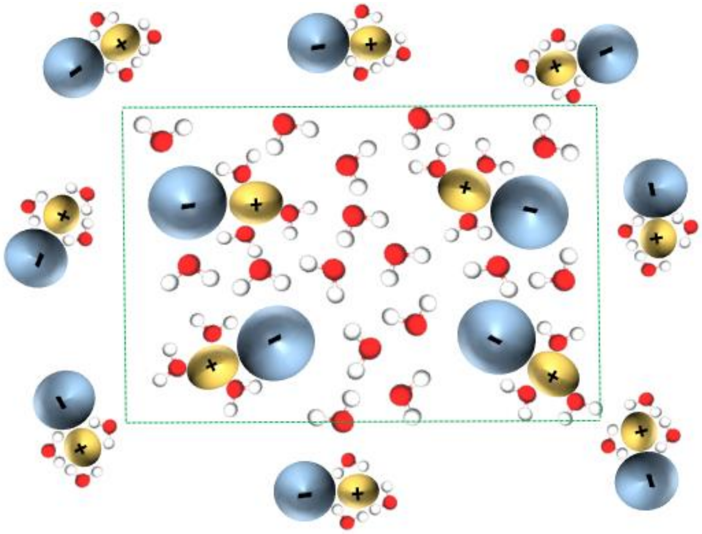

f. $x_s < x < x_{e2}$

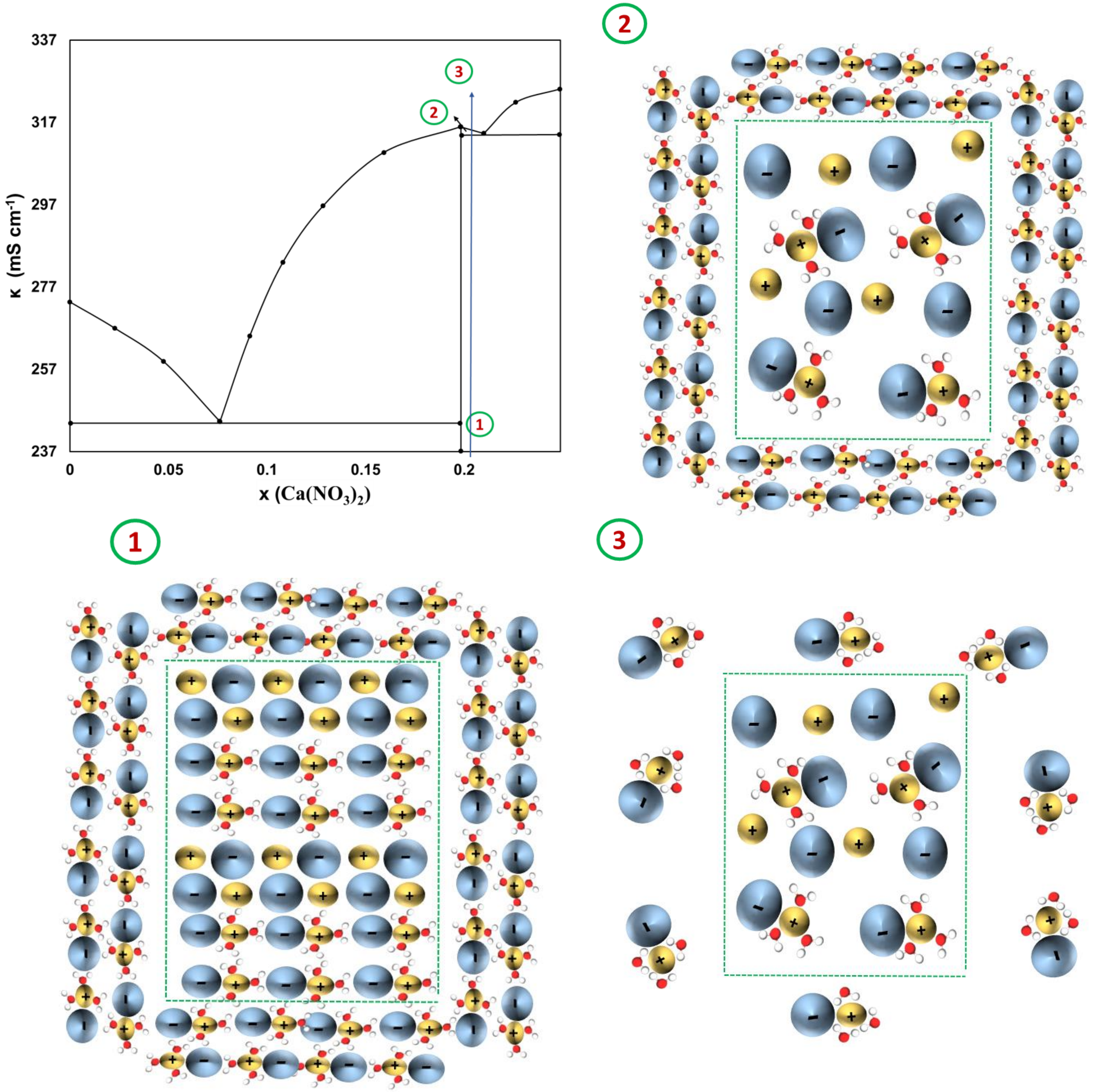

g. $x > x_{e2}$

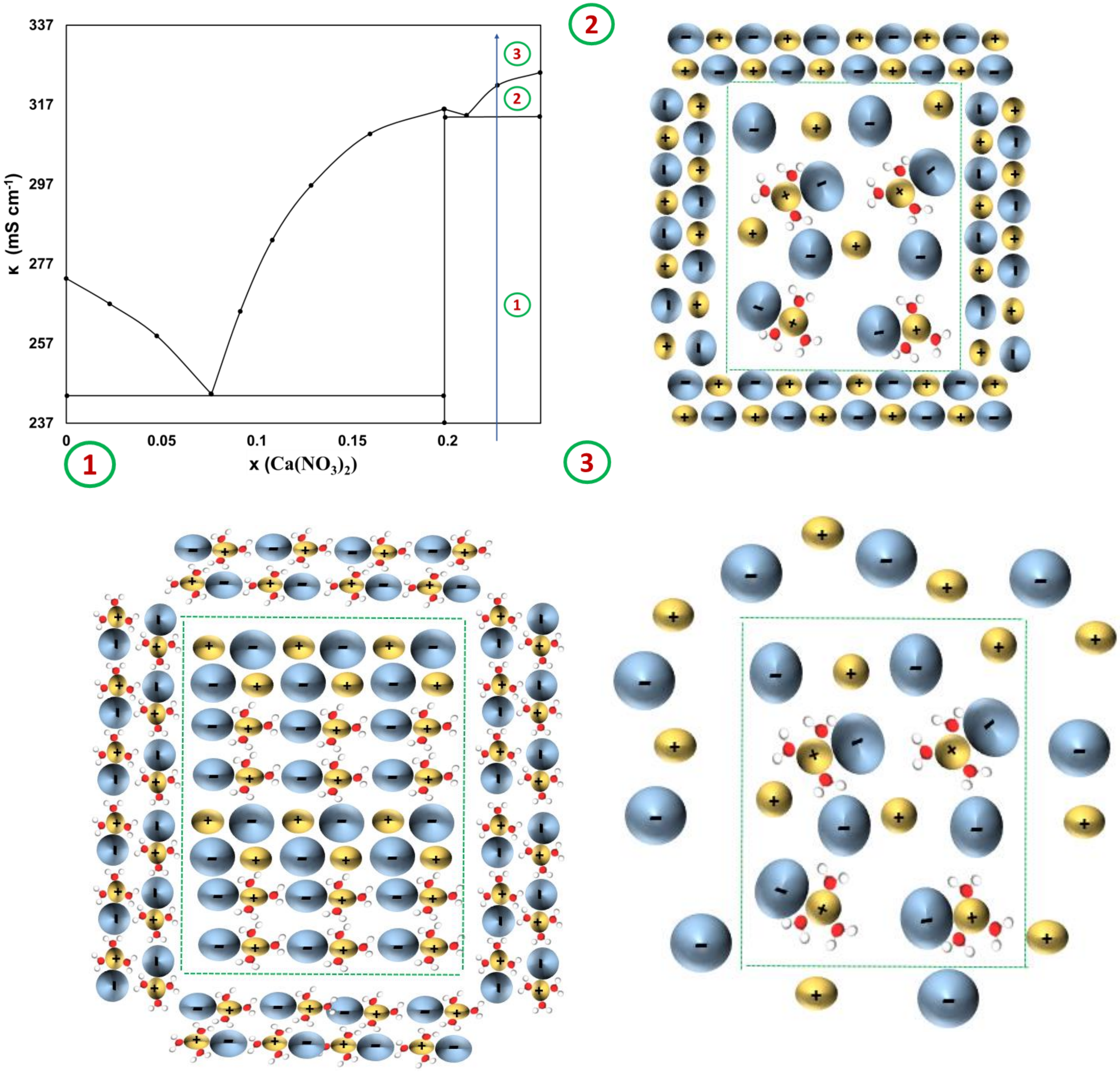

Supplement: Supplementary file 2 — Supplementary information [file 42004_2023_993_MOESM2_ESM.pdf]
